# Influence of the *ABCG2* gout risk 141 K allele on urate metabolism during a fructose challenge

**DOI:** 10.1186/ar4463

**Published:** 2014-01-30

**Authors:** Nicola Dalbeth, Meaghan E House, Gregory D Gamble, Bregina Pool, Anne Horne, Lauren Purvis, Angela Stewart, Marilyn Merriman, Murray Cadzow, Amanda Phipps-Green, Tony R Merriman

**Affiliations:** 1 Department of Medicine, Faculty of Medical and Health Sciences, University of Auckland, 85 Park Rd, Grafton, Auckland, New Zealand; 2 Department of Biochemistry, University of Otago, Dunedin, New Zealand

## Abstract

**Introduction:**

Both genetic variation in ATP-binding cassette sub-family G member 2 (*ABCG2*) and intake of fructose-containing beverages are major risk factors for hyperuricemia and gout. This study aimed to test the hypothesis that the *ABCG2* gout risk allele 141 K promotes the hyperuricaemic response to fructose loading.

**Methods:**

Healthy volunteers (n = 74) provided serum and urine samples immediately before and 30, 60, 120 and 180 minutes after ingesting a 64 g fructose solution. Data were analyzed based on the presence or absence of the *ABCG2* 141 K gout risk allele.

**Results:**

The 141 K risk allele was present in 23 participants (31%). Overall, serum urate (SU) concentrations during the fructose load were similar in those with and without the 141 K allele (*P*_SNP_ = 0.15). However, the 141 K allele was associated with a smaller increase in SU following fructose intake (*P*_SNP_ <0.0001). Those with the 141 K allele also had a smaller increase in serum glucose following the fructose load (*P*_SNP_ = 0.002). Higher fractional excretion of uric acid (FEUA) at baseline and throughout the fructose load was observed in those with the 141 K risk allele (*P*_SNP_ <0.0001). However, the change in FEUA in response to fructose was not different in those with and without the 141 K risk allele (*P*_SNP_ = 0.39). The 141 K allele effects on serum urate and glucose were more pronounced in Polynesian participants and in those with a body mass index ≥25 kg/m^2^.

**Conclusions:**

In contrast to the predicted responses for a hyperuricemia/gout risk allele, the 141 K allele is associated with smaller increases in SU and higher FEUA following a fructose load. The results suggest that *ABCG2* interacts with extra-renal metabolic pathways in a complex manner to regulate SU and gout risk.

**Clinical Trials Registration:**

The study was registered by the Australian Clinical Trials Registry (ACTRN12610001036000).

## Introduction

Genetic variation in ATP-binding cassette sub-family G member 2 (*ABCG2)* is a major risk factor for hyperuricemia and gout [1-3]. This gene encodes a high-capacity urate exporter [2,3], expressed in the intestine, liver and renal tubule [4]. The minor allele of *rs2231142* (141 K), that reduces efflux of uric acid by reducing expression of ABCG2 [5], is strongly associated with increased risk of hyperuricemia and gout [2,3,6,7]. Although ABCG2 is thought to control efflux of uric acid from the renal tubular cell into the lumen, a recent study has shown that dysfunction of ABCG2 results in increased renal uric acid excretion [8]. Furthermore, *Abcg2*-knockout mice have increased serum urate concentrations, increased renal uric acid excretion and reduced intestinal uric acid excretion [8]. These findings have led to the concept that ABCG2 expression in extra-renal tissues influences the effects of *ABCG2* genetic variation on gout risk, with the 141 K allele promoting extra-renal under-excretion and increased urinary excretion of uric acid [8].

Ingestion of fructose is also strongly associated with development of gout and hyperuricemia. In the Health Professional Follow-up Study, the multivariate relative risk for development of gout was 1.81 for those ingesting the highest quintile of fructose (>11.8% energy/day) [9] and each daily serving of sugar-sweetened beverage increases the risk of prevalent gout by 13% [10]. Similarly, in the National Health and Nutrition Examination Survey (NHANES) III, the multivariate odds ratio for hyperuricemia was 4.11 for those ingesting the highest quintile of fructose intake (>75 g/day), compared with the lowest quintile of <10 g/day [11]. Intervention studies have shown that fructose ingestion can rapidly increase serum urate concentrations [12-14]. This has been attributed to hepatic metabolism of fructose, which induces rapid depletion of ATP and increased urate production by degradation of preformed purine nucleotides [15]. It is possible that fructose also influences urate release from the hepatocyte into the systemic circulation and renal excretion of uric acid [10,16]. Of particular relevance to the current study is the observation that fructose-induced hyperuricemia is accentuated in patients with gout and first-degree relatives of patients with gout. In a small study of fructose-induced hyperuricemia, increases in serum urate were significantly higher in children of those with gout compared with healthy controls without a family history of gout [12], suggesting that fructose-induced hyperuricemia has a genetic component. We have recently reported that variation in the other major genetic risk factor for gout, solute carrier family 2, facilitated glucose transporter member 9 (*SLC2A9*), influences serum urate and fractional excretion of uric acid (FEUA) responses to a fructose load [17]. As *ABCG2* and *SLC2A9* are the two major genetic risk factors for gout [7], we wished to examine whether variation in *ABCG2* also contributes to serum urate responses to a fructose load. The aim of this study was to test the hypothesis that the *ABCG2* gout risk allele 141 K also promotes the serum urate response to fructose loading.

## Methods

This acute intervention study was designed to examine the influence of genetic variants on acute fructose-induced hyperuricemia in healthy participants. The methods of this study have been previously reported in detail [17]. Analysis of *ABCG2* was prespecified. Exclusion criteria were: gout, diabetes mellitus or fructose intolerance, diuretic use, fasting glucose >6 mmol/L. The study was approved by the New Zealand Multiregional Ethics Committee, and each participant gave written informed consent.

Following an overnight fast, participants consumed a sugar solution between 8 am and 9 am, and blood was obtained prior to ingestion and then 30 minutes, 60 minutes, 120 minutes, and 180 minutes after ingestion. Urine was obtained at each time point for testing of urate and creatinine. Weight and height were measured at the start of the study visit, and body mass index (BMI) was calculated. The sugar solution of 300 kcal/300 ml was consumed within 10 minutes, according to the protocol of Akhaven and Anderson for fructose-induced hyperuricemia [13]. This solution contained 80% fructose and 20% glucose (64 g fructose and 16 g glucose). Higher proportions of fructose, even at low concentrations, are poorly tolerated due to side effects of nausea and diarrhoea. The addition of glucose reduces these side effects [13].

Serum and urine chemistry was measured using the Roche Cobas c702 analyzer (Roche Diagnostics, Basel, Switzerland). L-Lactate and fructose concentrations in the 60-minute serum samples were measured using fluorescence-based assays (both Cayman Chemical Company, Ann Arbor, MI, USA) according to the manufacturer’s instructions. This time point was chosen as both serum lactate and fructose concentrations peak 60 minutes after intake of an oral fructose load [16]. Genotyping of the single nucleotide polymorphism (SNP) *rs2231142* was done using TaqMan SNP genotyping assay technology (Applied Biosystems, Carlsbad, CA) [6]. Genotyping was performed on 20 ng of genomic DNA using predesigned TaqMan® SNP drug metabolism assays (Life Technologies, Carlsbad, CA, USA) at 10× probe concentration, with ABsolute QPCR Rox Mix (Thermo Fisher Scientific, Waltham, M, USA) in a final reaction volume of 5 uL. The primer and probe sequences remain the proprietary information of Life Technologies. However, the context sequence for *rs2231142* (assay ID C__15854163_70) is (VIC/FAM) GCAAGCCGAAGAGCTGCTGAGAACT(G/T)TAAGTTTTCTCTCACCGTCAGAGTG. PCR was undertaken following the instructions of Thermo Fisher Scientific, specifically one enzyme activation cycle (95°C, 15 minutes) followed by 40 cycles of denaturation (95°C, 15 seconds) and annealing/extension (60°C, 1 minute). Assays were run on a Lightcycler® 480 Real-Time Polymerase Chain Reaction System (Roche, Indianapolis, IN, USA), and analyzed using the Lightcycler® 480 software. Successful genotyping data were available for 74 participants: 25 European Caucasian, 23 Eastern Polynesian (New Zealand Māori and Cook Island Māori) and 26 Western Polynesian (Samoan, Tongan, and Niuean) participants, and are included in the analysis. The Q126X SNP *rs72552713* was also genotyped using the Taqman assay as described above (the context sequence for *rs72552713* (assay ID C__98388180_20) is [VIC/FAM] AATGCAAACCCACTAATACTTACTT[G/A]TACCACGTAACCTGAATTACATTTG). All participants were homozygous for the major G allele. Therefore, no further analysis is presented for this variant.

The analysis plan specified change in serum urate as the primary endpoint. The key secondary endpoint was change in fractional excretion of uric acid (FEUA; the ratio between the renal clearance of uric acid to the renal clearance of creatinine, expressed as a percentage). Sample size calculations were based on the previous study of fructose-induced hyperuricemia by Stirpe *et al.*[12] This study showed significant differences between healthy volunteers with and without a family history of gout with 5 to 6 participants per group (all of European Caucasian ancestry). We selected the 141 K gout risk allele for analysis, and assumed that approximately 30% of participants would have the gout risk allele, based on our previous work [6]. In the smallest group comparison, n = 7 versus n = 18 within an ancestral subgroup, there was in excess of 90% power (at the 5% significance level for a two-tailed test) to detect a difference in the change in serum urate 120 minutes after an oral fructose challenge of at least the magnitude seen in the study by Stirpe *et al.*[12]. Sample size calculations were performed using PASS 2002 (Hintze, J (2006) Kaysville, UT, USA).

Data are presented as mean (SD) or n (%) for descriptive purposes; however, measures of effect are presented with the appropriate 95% CI. The primary analysis was a comparison of the hyperuricemic response to a fructose load in the entire group (n = 74), based on the presence or absence of the 141 K gout risk allele. Secondary and exploratory analyses were comparison of the fractional excretion of uric acid following a fructose load in the entire group, based on the presence or absence of the 141 K gout risk allele, and comparison of the hyperuricemic response to a fructose load based on subgroup analysis (within each ancestral subgroup).

Data were analyzed using a mixed models approach to repeated measures. Significant main and interaction effects were explored using the method of Tukey. Where indicated, sex and ancestry were adjusted for within the models. For change in serum urate and other biochemical variables, a mixed models analysis of covariance (ANCOVA) approach to repeated measures was used. For ANCOVA, the dependent variable was change from baseline, and baseline level was included as a covariate. Differences in participant characteristics between those with and without the 141 K gout risk allele were analyzed using the *t*-test for normally distributed data and Fisher’s exact test. All analyses were performed using SAS (SAS Institute Inc v 9.2). *P* <0.05 was considered significant and all tests were two-tailed.

## Results

### Participant characteristics

The 141 K risk allele was present in 23 participants (31%). There were 7 (9%) participants who were homozygous for the risk allele and 16 (22%) who were heterozygous for the risk allele. The baseline characteristics for those with and without the 141 K gout risk allele are shown in Table 1. There were more males in the group with the risk allele (*P* = 0.023). In all subsequent analysis, sex was included as a covariate in the mixed models analysis. The 141 K allele was present in only 4/23 Eastern Polynesian participants, and for this reason the Western and Eastern Polynesian groups were pooled into a single Polynesian group for the subsequent analysis. Unadjusted baseline serum urate concentrations were higher in the group with the 141 K allele. However, there was no difference in baseline serum urate concentrations between groups after adjusting for sex. Unadjusted baseline FEUA values did not differ between the two groups, but after adjusting for sex, baseline FEUA values were higher in the participants with the 141 K risk allele.

**Table 1:** Participant characteristics at baseline

	**Risk allele present, n = 23**	**Risk allele absent, n = 51**	***P***
Age, years	26.8 (13.6)	32.9 (16.5)	0.13
Male sex, n (%)	18 (78%)	25 (49%)	0.023
Body mass index, kg/m^2^	26.9 (4.9)	27.8 (6.0)	0.56
Waist circumference, cm	93.0 (11.7)	92.7 (17.9)	0.94
Systolic blood pressure, mmHg	127 (14)	127 (18)	0.99
Diastolic blood pressure, mmHg	70 (8)	72 (10)	0.44
Serum urate	0.41 (0.12) mmol/L	0.34 (0.1) mmol/L	0.018
	(6.8 (2.0) mg/dL)	(5.7 (1.7) mg/dL)	
Serum urate, adjusted for sex	0.37 (0.09) mmol/L	0.34 (0.09) mmol/L	0.21
	(6.2 (1.5) mg/dL)	(5.7 (1.5) mg/dL)	
Serum creatinine, mmol/L	0.084 (0.013)	0.077 (0.016)	0.088
Fractional excretion of uric acid, %	6.25 (2.86)	5.62 (1.78)	0.26
Fractional excretion of uric acid, % adjusted for sex	6.61 (2.06)	5.4 (2.07)	0.031
Serum glucose, mmol/L	4.69 (0.38)	4.69 (0.45)	0.99
Māori or Pacific ancestry, n (%)	16 (70%)	33 (65%)	0.79
Eastern Polynesian, n (%)	4 (17%)	19 (37%)	0.11
Western Polynesian, n (%)	12 (52%)	14 (27%)	0.064
European ancestry, n (%)	7 (30%)	18 (35%)	0.79

Unless specified, data are presented as mean (SD).

### The effect of ABCG2 genotype on biochemical responses during a fructose load in the entire group

**Figure 1:**
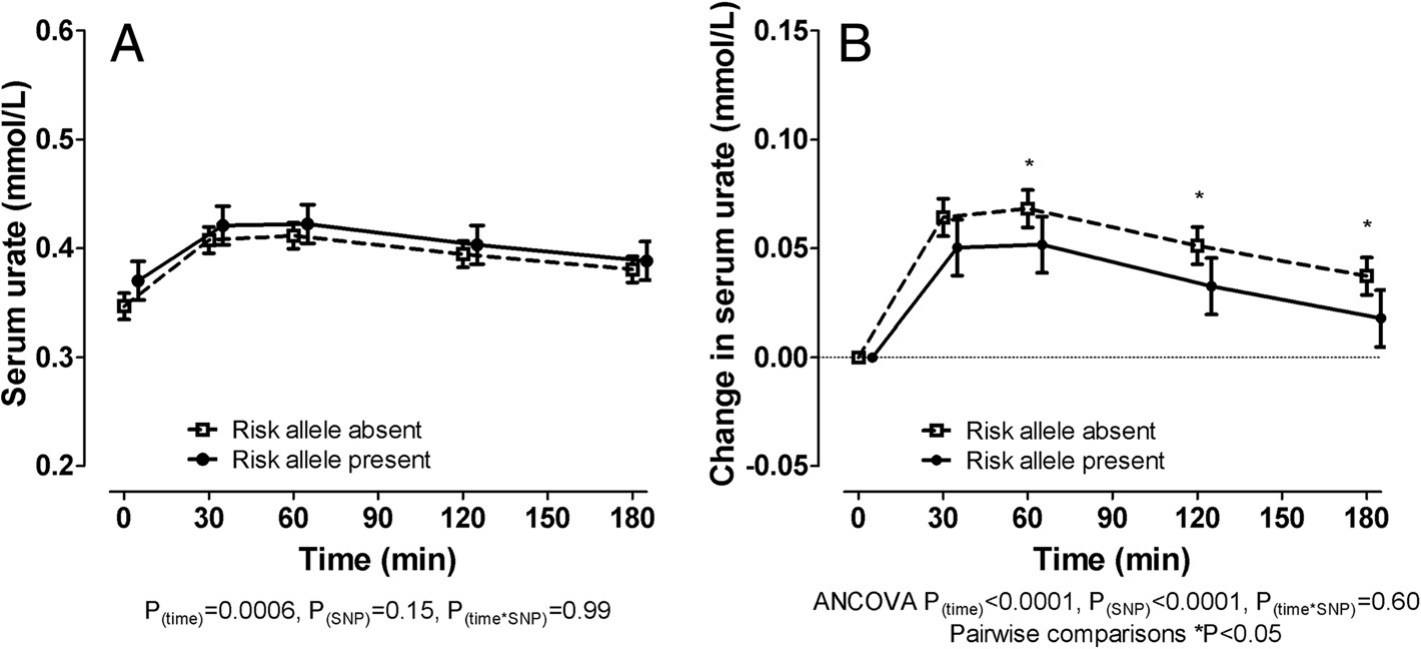
**Effect of *ABCG2 *genotype on serum urate concentrations during a fructose load in the entire group. (A)** Serum urate concentration. **(B)** Change in serum urate concentration. Data are presented as mean (95% CI). Sex and ancestry-adjusted graphs and *P*-values are shown throughout.

Overall, serum urate concentrations during the fructose load were similar in those with and without the 141 K allele (*P*_SNP_ = 0.15) (Figure 1A). However, the presence of the 141 K allele was associated with a smaller increase in serum urate following fructose intake (*P*_SNP_ <0.0001) (Figure 1B). This effect was observed after 60 minutes and persisted throughout the study period. A similar result was observed in the change in serum urate when only the male participants were included in the analysis (n = 43, *P*_SNP_ = 0.0001).

Separation of those with the risk allele into heterozygous and homozygous for the risk allele showed that both groups were associated with a similar smaller increase in serum urate following fructose intake compared with the group without the risk allele, but that there was no significant difference in the urate response between the heterozygous and homozygous groups (mixed models ANCOVA sex- and ancestry-adjusted *P*_genotype_ = 0.0003; post hoc tests none versus heterozygous for risk allele, *P* = 0.0006; none versus homozygous for risk allele, *P* = 0.07; heterozygous versus homozygous for risk allele, *P* = 0.95). Given the lack of difference between the heterozygous and homozygous risk allele groups and the small numbers of participants who were homozygous for the risk allele, the remaining analysis was done as presence versus absence of the risk allele.

**Figure 2:**
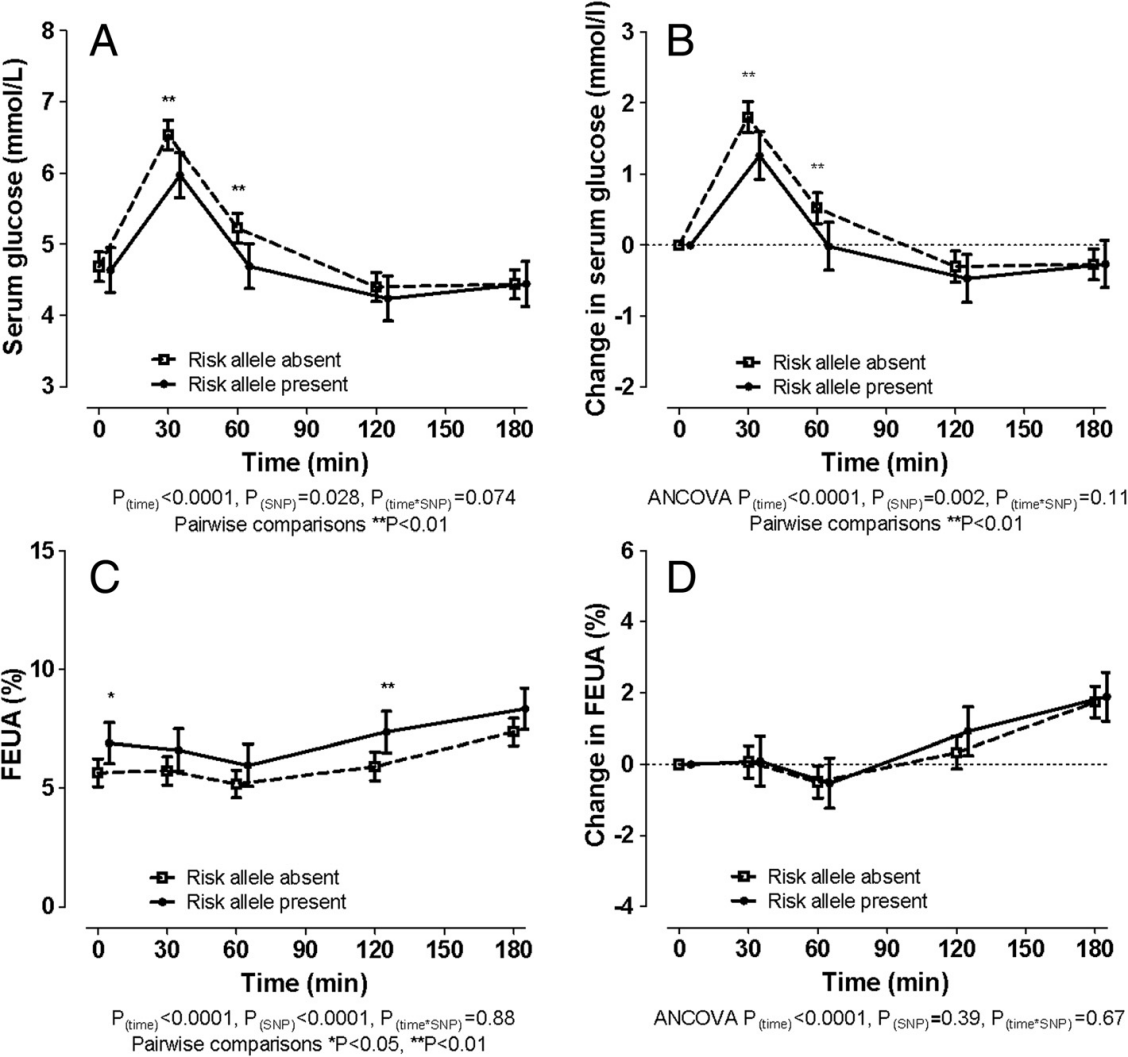
**The effect of *ABCG2 *genotype on serum glucose and fractional excretion of uric acid (FEUA) during a fructose load in the entire group. (A)** Serum glucose concentration. **(B)** Change in serum glucose concentration. **(C)** FEUA. **(D)** Change in FEUA. Data are presented as mean (95% CI). Sex- and ancestr-adjusted graphs and *P*-values are shown throughout. ANCOVA, analysis of covariance.

Those with the 141 K allele also had a lower serum glucose and smaller increase in serum glucose following the fructose load (*P*_SNP_ = 0.002) (Figure 2A and 2B). Serum lactate and fructose concentrations were not different between those with and without the 141 K allele 60 minutes after the fructose intake (sex- and ethnicity-adjusted *P* = 0.91 and 0.21 respectively, Additional file 1: Figure S1). Higher FEUA at baseline and throughout the fructose load was observed in those with the 141 K allele (*P*_SNP_ <0.0001) (Figure 2C). However, the change in FEUA in response to fructose was not different in those with and without the 141 K allele (*P*_SNP_ = 0.39) (Figure 2D).

### The effect of ABCG2 genotype on biochemical responses during a fructose load in ancestral subgroups: prespecified subgroup analysis

**Figure 3:**
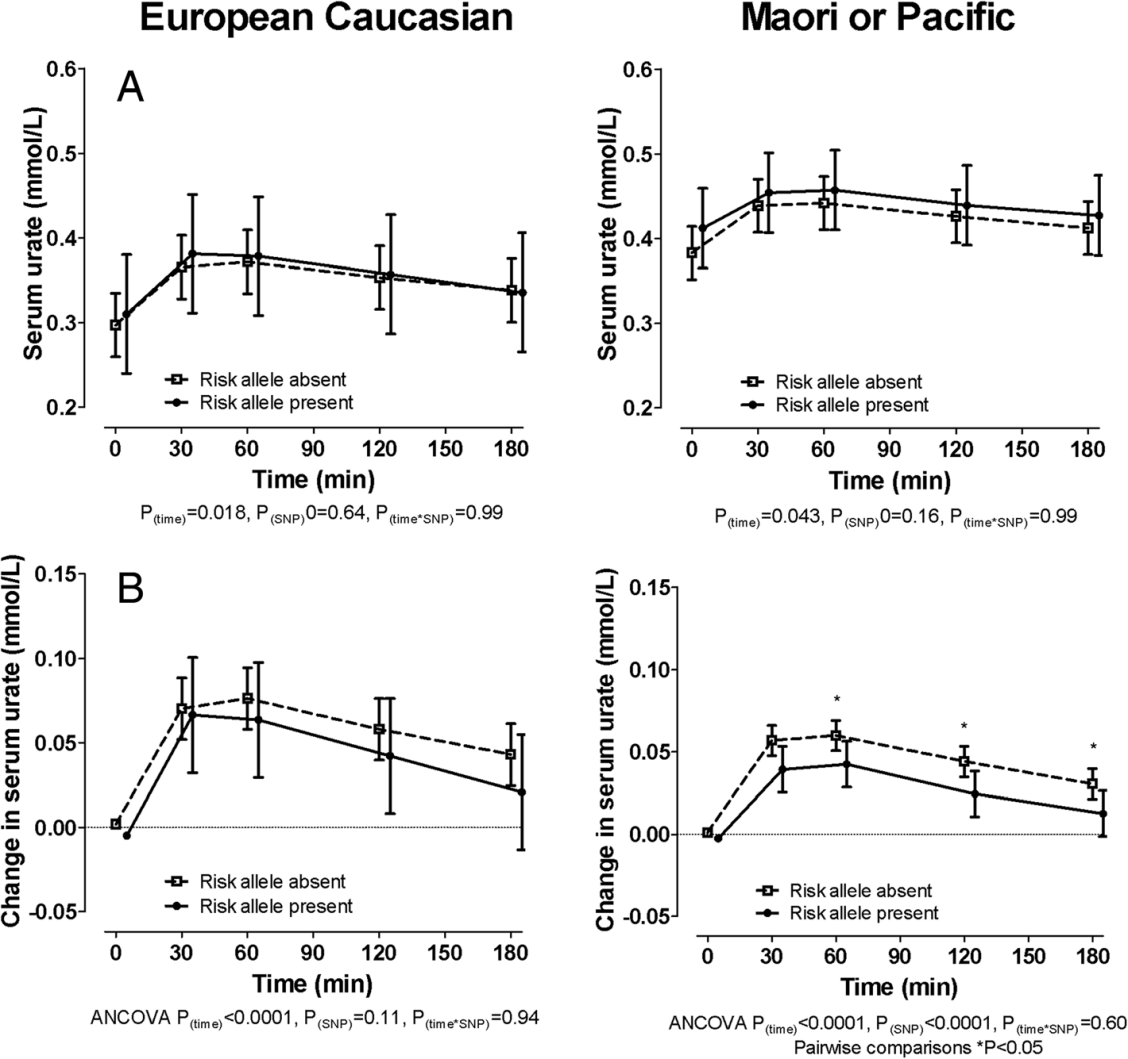
**The effect of *ABCG2 *genotype on serum urate concentrations during a fructose load in the ancestral subgroups. (A)** Serum urate concentration. **(B)** Change in serum urate concentration. Unless stated, data are presented as mean (95% CI). Sex- and ancestry-adjusted graphs and *P*-values are shown throughout. Left panel shows European Caucasian subgroup, right panel shows Māori and Pacific subgroup. Data are presented as mean (95% CI). Sex-adjusted graphs and *P*-values are shown throughout. ANCOVA, analysis of covariance.

In both the European Caucasian and Polynesian ancestral subgroups, serum urate concentrations during fructose challenge were similar in those with and without the 141 K allele (Figure 3A). The presence of the 141 K allele was associated with a smaller increase in serum urate concentrations following a fructose challenge in the Polynesian ancestral subgroup, with a similar trend in the European Caucasian ancestral subgroup (Figure 3B).

**Figure 4:**
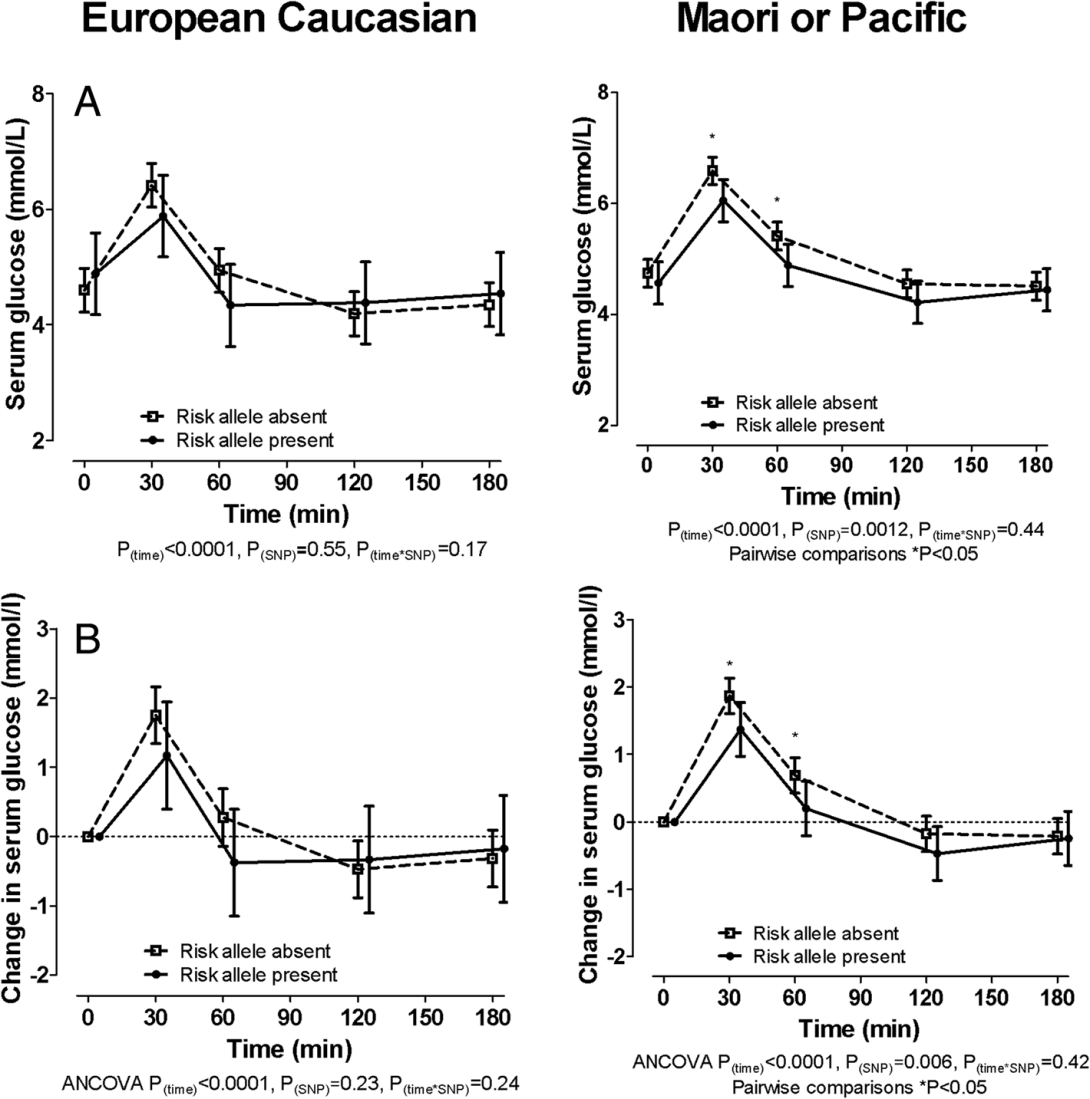
**The effect of *ABCG2 *genotype on serum glucose during a fructose load in the ancestral subgroups. (A)** Serum glucose concentration. **(B)** Change in serum glucose concentration. Data are presented as mean (95% CI). Left panel shows European Caucasian subgroup, right panel shows Māori and Pacific subgroup. Data are presented as mean (95% CI). Sex-adjusted graphs and *P*-values are shown throughout. ANCOVA, analysis of covariance.

**Figure 5:**
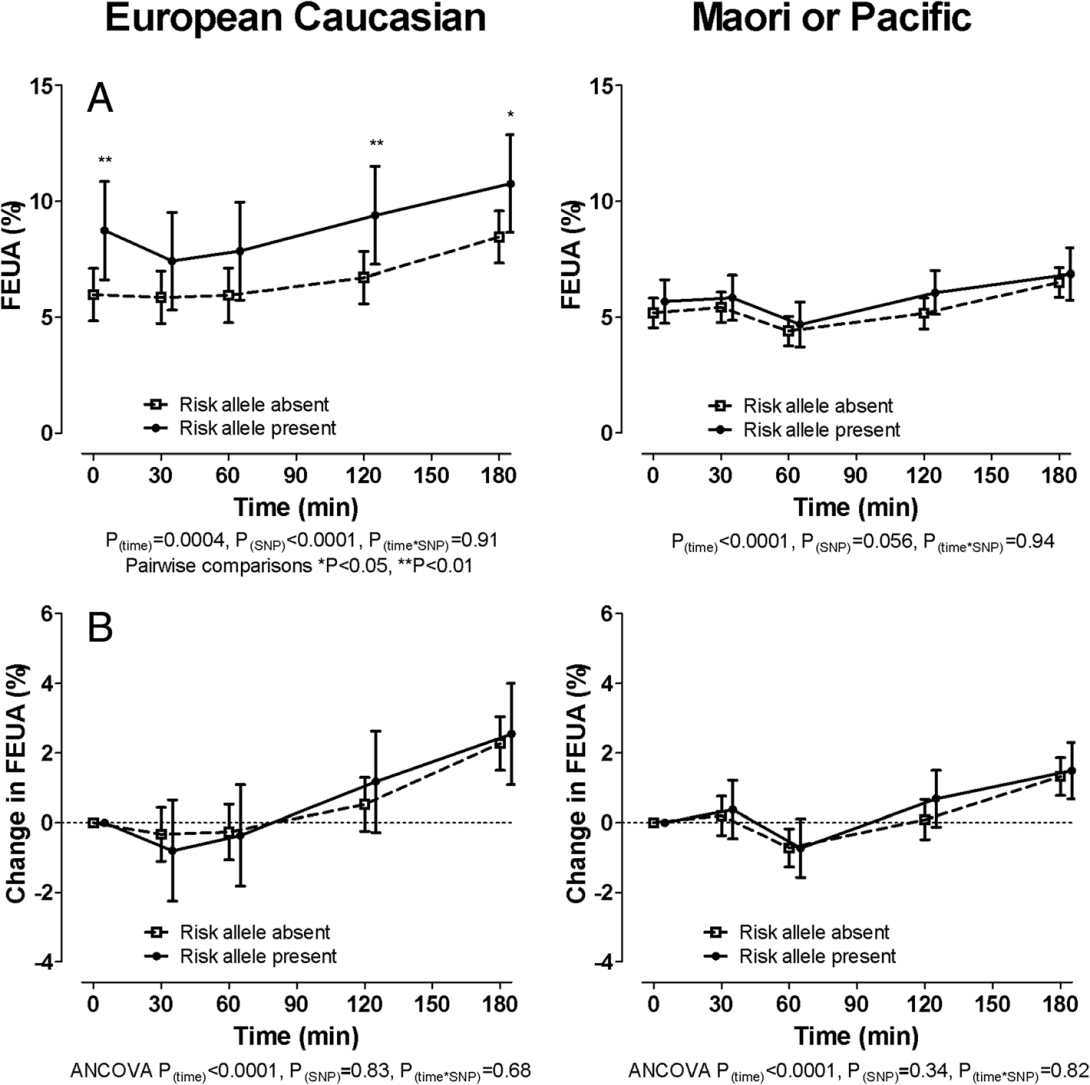
**The effect of *ABCG2 *genotype on fractional excretion of uric acid (FEUA) during a fructose load in the ancestral subgroups. (A)** FEUA. **(B)** Change in FEUA. Data are presented as mean (95% CI). Left panel shows European Caucasian subgroup, right panel shows Māori and Pacific subgroup. Data are presented as mean (95% CI). Sex-adjusted graphs and *P*-values are shown throughout. ANCOVA, analysis of covariance.

The 141 K allele was associated with lower serum glucose and smaller increase in serum glucose following a fructose challenge in the Polynesian subgroup, but not the European Caucasian subgroup (Figure 4). European Caucasian participants with the 141 K allele had higher fractional excretion of uric acid at baseline and following a fructose challenge than European Caucasian participants without the 141 K allele (Figure 5A). These associations were not significantly observed in the Polynesian ancestral subgroup (Figure 5A). The change in FEUA in response to fructose was not different in the two groups in either ancestral subgroup (Figure 5B).

### Additional subgroup analysis

There were 29 (39%) participants with hyperuricemia defined as serum urate concentrations ≥0.42 mmol/L. There was no difference in serum urate, FEUA or glucose responses to fructose between those with and without hyperuricemia (data not shown). In contrast, analysis subgroups based on BMI did show differences. There were 44 (59%) participants with BMI ≥25 kg/m^2^. The differences in change in serum urate and glucose were observed in those with BMI ≥25 kg/m^2^ (for urate, sex- and ethnicity-adjusted *P*_SNP_ = 0.003 and for glucose, sex- and ethnicity-adjusted *P*_SNP_ = 0.0006), but not in those with BMI <25 kg/m^2^ (for urate, sex- and ethnicity-adjusted *P*_SNP_ = 0.12 and for glucose, sex- and ethnicity-adjusted *P*_SNP_ = 0.77). Similar non-significant effects on change in FEUA were observed in those with BMI ≥25 kg/m^2^ and <25 kg/m^2^ (sex- and ethnicity-adjusted *P*_SNP_ = 0.36 and 0.79 respectively).

## Discussion

This study has highlighted the complex role of ABCG2 in regulation of serum urate concentrations. The *ABCG2* 141 K allele was associated with smaller increases in serum urate following a fructose load. This would be considered an unexpected response for a hyperuricemia/gout risk allele, and differs to that observed with *SLC2A9* where the presence of the protective allele is associated with a smaller change in serum urate compared to those without the protective allele [17].

In this study, the presence of the 141 K gout risk allele was also associated with lower glucose responses to the fructose load. The direction of change in serum glucose is similar to the direction of serum urate response, suggesting that the 141 K allele may influence both serum urate and glucose response to fructose. This could be related to an influence of Q141K on hepatic conversion of fructose to glucose, estimated to occur at a rate up to 60% depending on sex and health status [18]. The serum urate and glucose responses may be dependent or independent of each other. ABCG2 is not known to transport glucose or fructose. Furthermore, the lack of differences observed in serum lactate or serum fructose responses does not support the possibility that ABCG2 affects fructose absorption or degradation. The mechanism of this observation is currently unexplained, but could relate to ATP utilization by the ABCG2 alleles - the 141 K allele causes instability in the nucleotide binding domain and reduced protein expression [5]. Attempts to replicate this finding are warranted, particularly as other variants potentially influencing glucose metabolism have recently been associated with hyperuricemia in a large genome-wide association analysis [7].

Our data provide further support for the concept that the *ABCG2* 141 K allele does not increase hyperuricemia/gout risk through direct effects on renal tubular uric acid transport. Consistent with the report by Ichida *et al.* in which the 141 K risk allele was associated with increased urinary urate excretion in the Japanese population [8], we observed higher FEUA in participants with the risk allele both at baseline and after fructose loading, and no difference in the change in FEUA between the two groups. These results again contrast with our study of *SLC2A9*, in which the presence of the protective *SLC2A9* allele was associated with higher FEUA responses following a fructose load [17]. As ABCG2 is widely expressed in many tissues other than the renal tubule [4], it seems likely that the effects of *ABCG2* genetic variation on hyperuricemia and gout risk act on other sites of urate metabolism or clearance, most likely gastrointestinal [8]. It is possible that various uric acid transporters may influence the function of other transporters such as ABCG2, resulting in net increases or reductions in uric acid excretion. Larger studies are required to replicate the current results and also explore such interactions between transporters.

This work has demonstrated population-specific differences in the genotype-specific biochemical responses to fructose. In this study, the 141 K allele effects on change in serum urate and glucose were more pronounced in the Māori and Pacific ancestral subgroup. This effect may be in part related to the effects of BMI; as for *SLC2A9*[17], the effects of *ABCG2* genotype on serum urate response were primarily observed in people with high BMI. However, the significantly increased FEUA associated with the 141 K allele in the European Caucasian subgroup was not observed in the Māori and Pacific ancestral subgroup. Māori and Pacific people have high rates of early onset, severe hyperuricemia and gout [19], primarily due to reduced renal uric acid excretion [20,21]. The *ABCG2* FEUA results are consistent with the previous *SLC2A9* fructose challenge analysis, in which the *SLC2A9*-specific FEUA responses to fructose were observed in the European Caucasian subgroup, but not in the Māori and Pacific ancestral subgroup [17]. Together, these data suggest that other factors (genetic or non-genetic) may override the effects of genetic variants common to European Caucasian and Polynesian populations in renal uric acid handling in Polynesian people. Identification of these effects will be important to understand the basis of hyperuricemia in these high-risk groups.

## Conclusions

In contrast to the predicted responses for a hyperuricemia/gout risk allele, the 141 K allele is associated with smaller increases in serum urate and higher FEUA following a fructose load. There are also population-specific differences in the genotype-specific biochemical responses to fructose. The results suggest that ABCG2 interacts with extra-renal metabolic pathways in a complex manner to regulate serum urate and gout risk.

## Abbreviations

ABCG2: ATP-binding cassette sub-family G member 2; ANCOVA: analysis of covariance; BMI: body mass index; FEUA: fractional excretion of uric acid; SLC2A9: solute carrier family 2, facilitated glucose transporter member 9; SNP: single nucleotide polymorphism; SU: serum urate.

## Competing interests

The authors have no competing interest to declare.

## Authors’ contributions

ND (the guarantor) accepts full responsibility for the work and the conduct of the study, had access to the data, and controlled the decision to publish. ND conceived of the study, contributed to the data interpretation, and drafted the manuscript. MEH, AH, LP, and AS recruited participants and coordinated study visits. MEH also managed clinical data entry. BP completed laboratory testing. MM, MC and APG contributed to data acquisition and genetic data entry. GDG analyzed the data. TM conceived of the study, contributed to the data interpretation, and drafted the manuscript. All authors read and approved the final manuscript.

## Supplementary Material

Additional file 1: Figure S1 The effect of *ABCG2* genotype on serum fructose and lactate concentrations 60 minutes following fructose load. Data are presented as mean (SD). Click here for file
