# Supplementary figures and images for: Influence of the ABCG2 gout risk 141 K allele on urate metabolism during a fructose challenge
Source: Arthritis Res Ther. 2014 Jan 30;16(1):R34. doi: 10.1186/ar4463 (PMC3978630; doi:10.1186/ar4463)

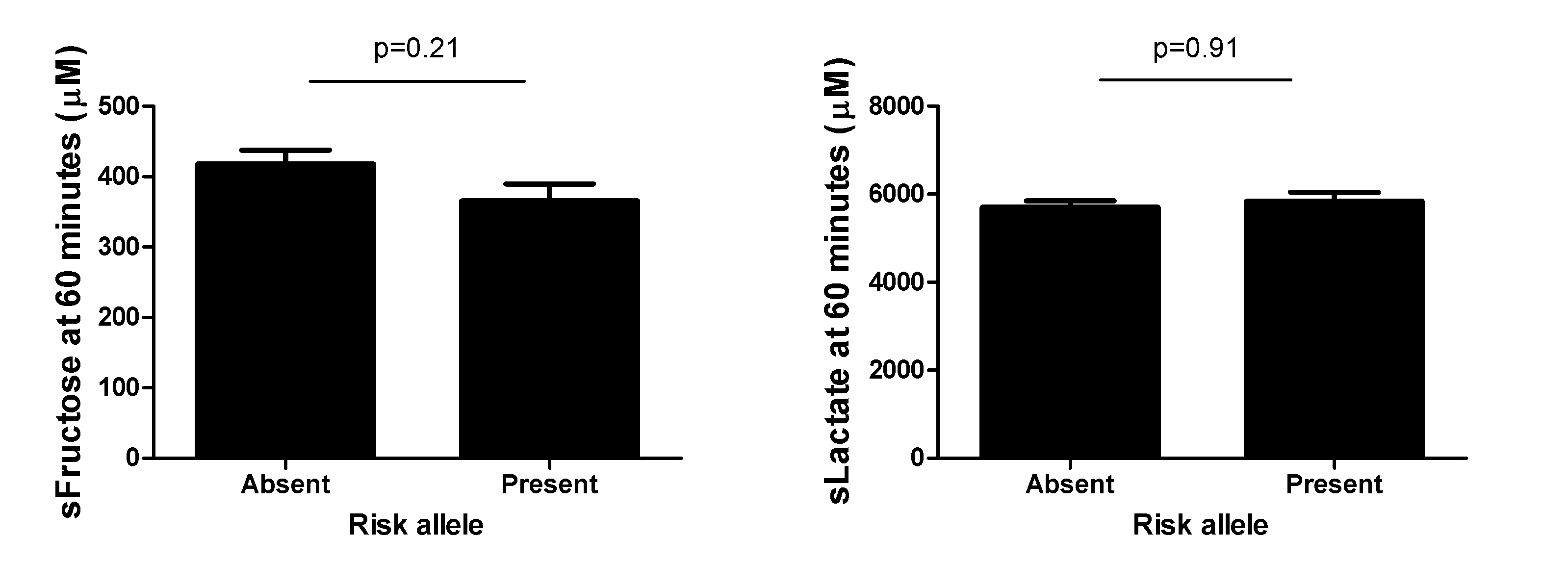

Supplement: Additional file 1: Figure S1 — The effect of ABCG2 genotype on serum fructose and lactate concentrations 60 minutes following fructose load. Data are presented as mean (SD). [file ar4463-S1.jpeg]
